# NSAIDs beyond COX: *In Silico* and *In Vitro* Insights into Acetylcholinesterase Modulation

**DOI:** 10.1021/acsomega.5c08750

**Published:** 2025-11-13

**Authors:** Airam Roggero, Pedro Mathias Loyola, Caroline Ramos da Cruz, Willian Haniel Bezerra de Carvalho Santos, Paloma Peixoto Borges, Adeilso Bispo dos Santos Junior, Fábio Martins, Marcos A. Oliveira, Sérgio F. Sousa, Marcos H. Toyama

**Affiliations:** † LAQV/REQUIMTE—BioSIM, Department of Biomedicine, Medicine Faculty of Porto University, Porto District, Porto 4099-002, Portugal; ‡ BIOMOLPEP, Department of Biology, Institute of Biosciences UNESP/CLP, São Paulo 11330-900, São Vicente, Brazil; § Paulista University (UNIP), Institute of Sciences and Health, Department of Health, São Paulo 11075-110, Santos, Brazil; ∥ ABC Federal University (UFABC)—Postgraduate Studies in Biotechnoscience, Department of Biotechnology, São Paulo 9280-560, Santo André, Brazil; ⊥ LABIMEs Department of Biology, Institute of Biosciences UNESP/CLP, São Paulo 11330-900, São Vicente, Brazil; # Department of Chemical Engineering, Faculty of Science and Technology, University of Coimbra, Coimbra 3004-531, Portugal

## Abstract

Nonsteroidal anti-inflammatory drugs (NSAIDs) are widely prescribed for their cyclooxygenase (COX) inhibitory activity. However, mounting evidence suggests that their pharmacology extends beyond this canonical pathway. In this study, the potential of NSAIDs to modulate human acetylcholinesterase (AChE), a key enzyme linking neuroinflammation, metabolic dysfunction and degenerative diseases, was evaluated. An integrative strategy combining silicon and vitro approaches was employed. Structure-based molecular docking using GOLD, molecular dynamics simulations with AMBER21, and MM-GBSA free energy calculations were performed to assess binding stability and energetic favorability. Among the compounds that were tested, nimesulide displayed the highest binding affinity (Δ*G* = −24.2 kcal/mol), followed by dipyrone (−16.0 kcal/mol), ibuprofen (−11.0 kcal/mol), and paracetamol (−9.0 kcal/mol). The per-residue energy decomposition revealed the involvement of aromatic and polar residues in the catalytic gorge, displaying interaction patterns analogous to those of classical AChE inhibitors. Experimental enzymatic assays corroborated these predictions, demonstrating a dose-dependent inhibition of AChE activity by nimesulide at micromolar concentrations. These findings suggest that NSAIDs, particularly nimesulide, may act as dual modulators with both anti-inflammatory and cholinergic regulatory effects. This work underscores the translational potential of drug repurposing and highlights the importance of combining computational and biochemical methods to uncover novel therapeutic functions of established drugs.

## Graphical abstract

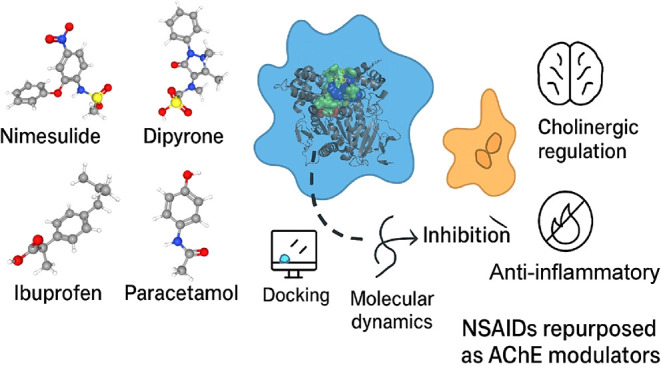

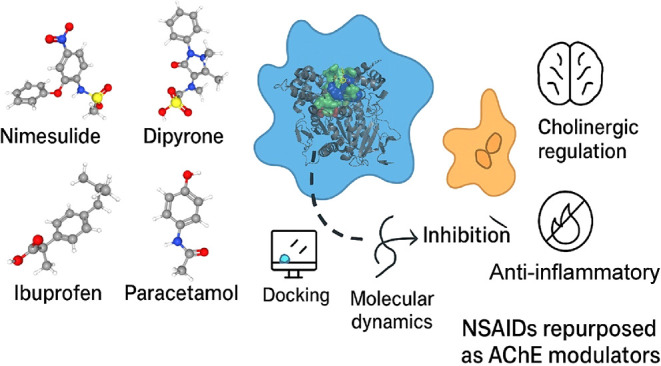

## 1 Introduction

While acute inflammation aims to eliminate harmful stimuli and restore tissue function, chronic inflammation is characterized by persistent immune activation, redox imbalance and altered lipid metabolism, collectively disrupting tissue homeostasis.^1^

Recent neuropsychiatric studies have demonstrated that, in addition to their classic effects on neurotransmitters, antidepressants modulate peripheral and central cytokine networks. This finding suggests the possibility of a more extensive pharmacological role for these compounds, extending beyond their initial serotonin target.^2^ In addition, it is proposed that nonsteroidal anti-inflammatory drugs (NSAIDs) may interact with alternative targets, such as AChE or sPLA_2_, which justifies the present multitarget screening.

The therapeutic effects of NSAIDs have long been attributed to the inhibition of cyclooxygenase (COX) enzymes, particularly the inducible COX-2 isoform, which catalyzes the conversion of arachidonic acid into pro-inflammatory prostaglandins. This reduces the production of eicosanoids, thereby alleviating symptoms such as pain, fever, and edema. However, the nonselective inhibition of COX-1 by many NSAIDs can lead to adverse effects on the gastrointestinal, renal, and cardiovascular systems due to its homeostatic functions in these tissues.^1,3^

In addition to modulating inflammation and cellular signaling, these agents may interfere with tumor progression by inducing apoptosis and inhibiting angiogenesis and metastasis. These effects correlate with the overexpression of COX-2 in many tumors and its association with malignancy, which further supports the need to explore the actions of NSAIDs beyond inflammation.^4–7^

Some studies have demonstrated that NSAIDs also affect other molecular targets related to lipid metabolism and cellular stress. Among these, phospholipase A2 (PLA2), lysophosphatidylcholine acyltransferase 3 (LPCAT3), and acetylcholinesterase (AChE) have gained prominence for their critical roles in phospholipid remodelling, inflammatory signaling, and maintenance of membrane integrity.^8–10^

While it is widely accepted that many NSAIDs exhibit limited permeability of the blood-brain barrier (BBB), in vitro transport studies have revealed that substances such as diclofenac and ibuprofen can permeate at rates influenced by membrane transporters.^2,11,12^ Furthermore, NSAIDs have been associated with central effects, such as the inhibition of β-amyloid aggregation and modulation of glial cells. This finding suggests the possibility of more extensive pharmacological actions by these compounds, extending beyond the scope of simple COX inhibition.

In order to ensure a representative evaluation, five NSAIDs with different predicted interaction profiles and BBB permeability behaviors were selected for analysis: nimesulide, diclofenac, ibuprofen, paracetamol and dipyrone. The selection comprised compounds with coverage values distributed across the highest, intermediate and lowest categories, thereby enabling robust comparative analysis. Although nimesulide has been associated with hepatotoxicity and clinical restrictions, it was retained in the analysis due to its high predicted affinity values, serving as a comparative reference for NSAID–AChE interactions rather than as a direct repurposing candidate.^13,14^

Moreover, the inclusion of molecules such as dipyrone and nimesulide, which have limited BBB permeability, is justified by the fact that AChE is widely distributed in peripheral tissues such as erythrocytes, platelets, and the liver, where it is involved in functions related to inflammatory regulation and oxidative stress. Consequently, even in conditions of minimal CNS penetration, these drugs can exert significant regulatory effects through the modulation of specific AChE. It is also important to emphasize that the classical affinity of NSAIDs toward COX remains predominant, and the potential modulation of AChE is likely to occur at higher or tissue-specific concentrations. Therefore, the findings presented here should be interpreted as mechanistic hypotheses that warrant further validation in cellular and *in vivo* models.^2,15,16^

PLA_2_ enzymes initiate the release of arachidonic acid and lysophospholipids from membrane phospholipids, thereby contributing to both prostaglandin synthesis and the Lands cycle. This remodelling, known as the Lands cycle, is a critical pathway involving LPCATs, particularly LPCAT3, which reacylates lysophosphatidylcholine (LPC) in order to restore membrane composition and integrity.^8,9^

Structurally, LPCAT3 contains conserved acyltransferase domains with essential residues such as serine, histidine and aspartate, which form a catalytic triad responsible for acyl group transfer. LPCAT3 activity modulates immune signaling, oxidative balance and macrophage polarization.^10,17^ Hydrophobic residues such as leucine and isoleucine promote membrane integration, while polar amino acids within the active site interface guide substrate orientation and transition state stabilization. NSAIDs can modulate this enzymatic axis: some directly inhibit PLA_2_, while others alter LPCAT activity, thereby influencing eicosanoid production and inflammatory resolution.^18^

Disruption of this lipid remodelling network impairs membrane homeostasis and contributes to chronic inflammation, as observed in obesity and cancer. Inflamed adipose tissue secretes cytokines, eicosanoids, and enzymes such as COX-2 and PLA_2_, promoting insulin resistance, angiogenesis, and immune evasion.^15,19^ Similarly, neurodegenerative diseases—including Alzheimer’s and Parkinson’s—share overlapping inflammatory signatures, such as microglial activation, cholinergic dysfunction, increased reactive oxygen species (ROS), and dysregulation of phospholipid metabolism.^20,21^

AChE plays a dual role in the central nervous system and in immune regulation. It terminates synaptic transmission by hydrolyzing acetylcholine (ACh), but its overexpression reduces ACh bioavailability and impairs the cholinergic anti-inflammatory pathway. This mechanism has been associated with several neuroinflammatory and neurodegenerative disorders.^22,23^ AChE isoforms also participate in apoptosis, cell adhesion and neurodevelopment, suggesting a broader role in immune-neural communication.^24^

Was selected the 4M0E structure for the treatment of a human AChE model in complex with the natural inhibitor dihydrotanshinone I, which occupies both the catalytic and peripheral sites. This characteristic is of particular relevance for docking studies, as it facilitates the evaluation of both classical and alternative binding interactions, including those analogous to those observed with clinical inhibitors such as donepezil. In addition, the 4M0E complex exhibits adequate crystallographic resolution (2.35 Å) and has been extensively utilized in previous studies examining AChE inhibition by natural and synthetic molecules, thereby reinforcing its validity as a reference structural model.^25^ The inhibitor under scrutiny is a natural compound, and its dimensions approximate those of the standardized inhibitor employed in screening trials. This renders it a more appropriate model for comparison.

Which collectively disrupt tissue homeostasis, LPCAT3 expression in the liver and adipose tissue directly links lipid remodelling to metabolic inflammation, supporting the notion that targeting this axis could have therapeutic benefits.^26^

When integrated with experimental validation, these computational strategies have the potential to accelerate drug repurposing efforts and deepen our understanding of disease-modifying mechanisms. The NSAIDs are promising candidates for therapeutic repurposing due to the shared inflammatory mechanisms across metabolic, oncological and neurodegenerative diseases.

The present study focuses on AChE as a key modulator of the cholinergic anti-inflammatory pathway and a promising non-COX molecular target for NSAIDs. The objective of this study is 2-fold: first, to employ computational modeling in order to elucidate the mechanisms of NSAID interaction with AChE; and second, to utilize experimental validation in order to explore their therapeutic potential in chronic inflammatory, metabolic, and neurodegenerative diseases.

## 2 Materials and Methods

### 2.1 In Silico Study

The chemical structures of NSAIDs were obtained from the PubChem database, while the target protein structures (LPCAT3 and AChE) were retrieved from the Protein Data Bank (PDB)^27^ The crystallographic structures of AChE (PDB ID: 4M0E)^25^ and LPCAT3 (PDB ID: 7F3X)^25^ were used as references for docking and redocking simulations, retaining the cocrystallized ligands to validate the docking protocol.

Molecular docking was performed using the Genetic Optimization for Ligand Docking (GOLD) software integrated with DataWarrior (DW). Docking simulations were focused on the catalytic sites of the target proteins. Protocol validation included redocking of cocrystallized ligands, with root-mean-square deviation (RMSD) values below 2.0 Å confirming the accuracy of the docking approach. Each ligand was subjected to 100 genetic algorithm runs to ensure comprehensive conformational sampling. This integrated methodology provided reliable predictions of ligand binding modes and affinities, supporting subsequent molecular dynamics simulations and free energy calculations.^28,29^

### 2.2 Molecular Docking and Redocking Validation

With GOLD the proteins were prepared by removing water molecules, adding hydrogen atoms, and assigning protonation states consistent with physiological pH, and for dockings we use the ChemPLP scoring function was used to rank the predicted binding affinities of the ligands.^29–31^

In order to validate the docking protocol, redocking of cocrystallized ligands was performed for all available structures, and with a library of the NSAIDs in multiple scores on GOLD to evaluate the stability. The root-mean-square deviation (RMSD) between the experimental and predicted poses was calculated, with values <2.0 Å confirming the reliability of the protocol. For proteins devoid of crystallographic inhibitors, reference ligands were obtained from the ChemBL database. A total of 100 genetic algorithm runs per ligand were utilized to ensure conformational sampling.^28,32^

This integrated approach enabled the prediction of the preferred orientation of the molecules in relation to the enzymes’ active sites This algorithm quantitatively evaluates factors such as the structural complementarity between the ligand and the active site, as well as interaction energies. This combined approach provided valuable insight into how each compound interacts with the active sites of the target enzymes. All figures related to docking were generated using PyMOL.^29–31^

### 2.3 Molecular Dynamics Simulations

The MD simulations were conducted utilizing the AMBER 21 software. Proteins were parametrized with the AMBER14SB force field, while NSAIDs was parametrized with the General AMBER Force Field (GAFF), using RESP charges derived from HF/6–31G­(d) quantum calculations. All systems were solvated in TIP3P water boxes with a minimum buffer of 15 Å. For membrane-associated targets (AchE and LPCAT3), simulations included embedding in a POPC lipid bilayer, reflecting a physiologically relevant environment. The addition of Na^+^ and Cl^–^ ions was undertaken in order to neutralize the system and adjust the ionic strength to 0.15 M.^33,34^

Energy minimization was performed in four stages: (1) water molecules, (2) hydrogens, (3) protein side chains, and (4) the full system, each for 10,000 steps. Equilibration was performed in both the NVT (Navier–Stokes-Taylor) and NPT (Navier–Stokes-Poiseuille) ensembles, with gradual heating to 310 K. Production runs of 100 ns were conducted in triplicate. The trajectories were analyzed using CPPTRAJ to calculate root-mean-square deviation (RMSD), and hydrogen bond stability.^33,34^

Energy minimization was performed in four stages: (1) water molecules, (2) hydrogens, (3) protein side chains, and (4) the full system, each for 10,000 steps. Equilibration was performed in both the NVT (Navier–Stokes-Taylor) and NPT (Navier–Stokes-Poiseuille) ensembles, with gradual heating to 310 K. Production MD runs of 200 ns were conducted in three replicates independent simulations, enhancing sampling and reproducibility.

In addition to residue-wise energy decomposition, heatmaps were generated to visualize the contributions of key residues qualitatively and quantitatively to protein–ligand interactions throughout the simulations. This facilitated identification of critical interaction sites and aided mechanistic interpretation.

The trajectories were analyzed using CPPTRAJ to calculate root-mean-square deviation (RMSD), and hydrogen bond stability.

The binding free energies (Δ*G*) were estimated using the MM-GBSA method, based on a minimum of 500 conformations extracted from equilibrated MD trajectories.^35^

A per-residue decomposition was conducted in order to ascertain the key residues that were contributing to binding. This approach is predicated on the premise that it accounts for protein flexibility and consequently overcomes the limitations that are inherent to static docking.

### 2.4 Enzymatic Screening

#### 2.4.1 Acetylcholinesterase Inhibitor Assay

The inhibitory activity of selected NSAIDs against AChE was evaluated using a commercial colorimetric kit (Sigma-Merck, Germany) according to manufacturer protocols. Measurements were performed in three replicates at 412 nm using Ellman’s method, with enzyme activity expressed as percentage inhibition relative to untreated control samples. Enzyme activity was measured and expressed as percentage inhibition relative to untreated controls.

#### 2.4.2 Phospholipase A2 Inhibitor Assay

The assessment of PLA2 activity was conducted in accordance with a protocol that had been previously established and adapted for use with 96-well plates. Reaction mixtures (260 μL) contained 10 mM Tris-HCl, 10 mM CaCl2, 100 mM NaCl (pH 8.0), 20 μL of substrate, and 20 μg of enzyme. Absorbance at 425 nm was measured at 10 min intervals over a 40 min period at a temperature of 37 °C. All experiments were conducted in three replicate biological and technical replicates. Median inhibitory concentrations (IC_50_) were calculated using nonlinear regression.

### 2.5 Statistical Analysis Data

The data are expressed as the mean ± standard deviation (SD). Statistical analysis was performed using GraphPad Prism 9.0. The statistical analysis comprised two-way ANOVA followed by Bonferroni post hoc tests, with statistical significance defined as *p* < 0.05.

## 3 Results and Discussion

A molecular docking analysis was performed using the Gold ChemPLP scoring function, in Table 1 in order to evaluate the interaction of commonly used nonsteroidal anti-inflammatory drugs with acetylcholinesterase (PDB: 4m0e). The reference inhibitor, dihydrotanshinone I (1YL),^25^ cocrystallized in the AChE structure, achieved the highest interaction score (81.10), thus serving as a benchmark for the normalization and comparative analysis of the tested ligands.

**Table 1:**
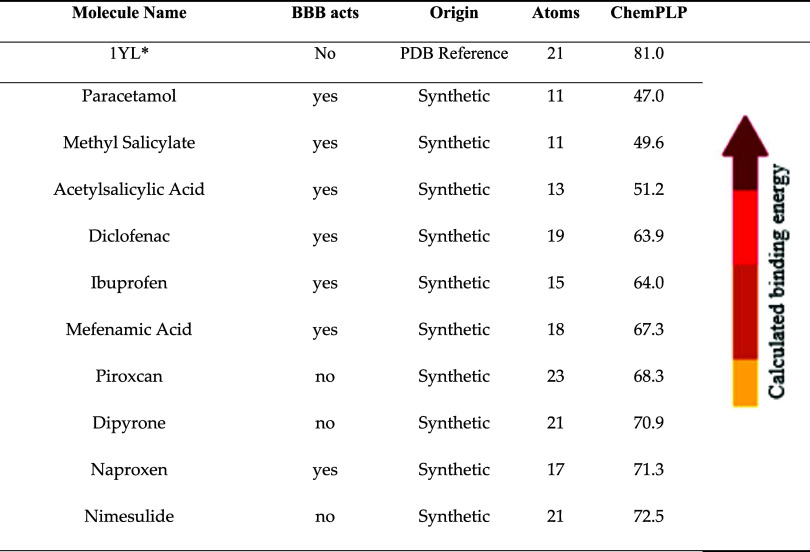
Gold PLP Interaction Analysis of Anti-Inflammatory Drugs with AchE Protein (PDB: 4m0e), Highlighting the Optimal Interaction Scores Achieved with Acetaminophen and Ibuprofen

* Dihydrotanshinone I is a reference inhibitor for the AchE protein, so a molecular docking was made to guide the results, and due to the values, a normalized score was created for better interaction analysis.^25^

Among the NSAIDs, nimesulide (NIM) exhibited the highest predicted affinity (73.56), suggesting strong binding to the AChE active site. This outcome is consistent with the polycyclic aromatic scaffold and lipoamides group of the compound, which facilitate favorable hydrophobic contacts and hydrogen bonds within the aromatic gorge of the enzyme. Such interactions may confer additional stability to the complex, thereby supporting the hypothesis that nimesulide could exert modulatory effects on AChE beyond its canonical COX inhibition, in line with reports of its pleiotropic actions in inflammatory and neurodegenerative contexts.^36^

In the second position among the drugs that were examined, Diclofenac (DCF) obtained an intermediate score of 62.59. The hypothesis that this interaction is stabilized by electrostatic contacts between the carboxylate moiety of diclofenac and basic residues at the catalytic site is one that merits further investigation. Although this result is less favorable than that obtained with nimesulide, the score indicates partial exploitation of the enzyme’s catalytic hotspots, which may explain the literature evidence describing diclofenac’s secondary effects on the central nervous system, unrelated to COX inhibition.^37^

The analysis revealed that Ibuprofen (IBU) exhibited a lower score (54.62), which was comparable to that of dipyrone (55.35). The relatively modest affinity can be attributed to the compact nature of ibuprofen, which limits its ability to fully occupy the deep aromatic gorge of AChE. However, the positive docking score suggests the potential for residual binding, which may contribute to off-target neurological effects associated with ibuprofen therapy.^37,38^

Conversely, acetaminophen (ACE) exhibited the lowest score (48.39) among the evaluated molecules, a finding consistent with its pharmacological mechanism of action, which does not involve direct AChE inhibition. The reduced predicted affinity of the subject is indicative of its limited role in cholinergic modulation, thus validating the docking approach in distinguishing ligands with weak binding potential.^39^

Taken together, these results suggest that while all tested NSAIDs display some degree of interaction with AChE, nimesulide emerges as the most promising candidate, followed by diclofenac, whereas ibuprofen and acetaminophen show weaker affinities. This raises the intriguing possibility that certain NSAIDs may exert noncanonical modulatory effects on AChE, a hypothesis that warrants further validation through in vitro enzymatic assays and cellular studies.^40,41^

Despite the brain penetration of some NSAIDs being limited, neuroimmunology models indicate that peripheral immunological alterations can modulate the brain environment via cytokine transport, endothelial activation, or the vagus nerve.^2^ Consequently, even peripheral AChE modulation may exert an indirect effect on relevant neural circuits.

It is important to highlight that, although compounds such as dipyrone and nimesulide have restricted or uncertain permeability to the blood-brain barrier, their ability to modulate AChE remains physiologically relevant. This is due to the fact that the enzyme is not confined to the CNS but also performs vital peripheral functions in the regulation of inflammation, oxidative stress, and lipid remodelling. This observation serves to reinforce the relevance of the selection of these compounds in the present screening, given that both the central and peripheral effects of AChE inhibition have the potential to contribute to the multitarget profile of NSAIDs.

The other protein target for this study was LPCAT3, using the PDB code 7F3X, which is essential in the Lands’ pathway, by regulating membrane phospholipid fatty acid composition, directly influencing membrane fluidity and functionality. This regulation is vital for cellular processes such as cell division, signaling, and molecule transport. LPCAT3 synthesizes phospholipids.^42^

In order to assess the binding affinities of NSAIDs to LPCAT3, the results were also obtained through the GOLD software’s PLP scoring algorithm. These metrics, presented in Table 2, offer insights into how NSAIDs might modulate this enzyme, impacting key biological functions (Figure 1).

**Figure 1:**
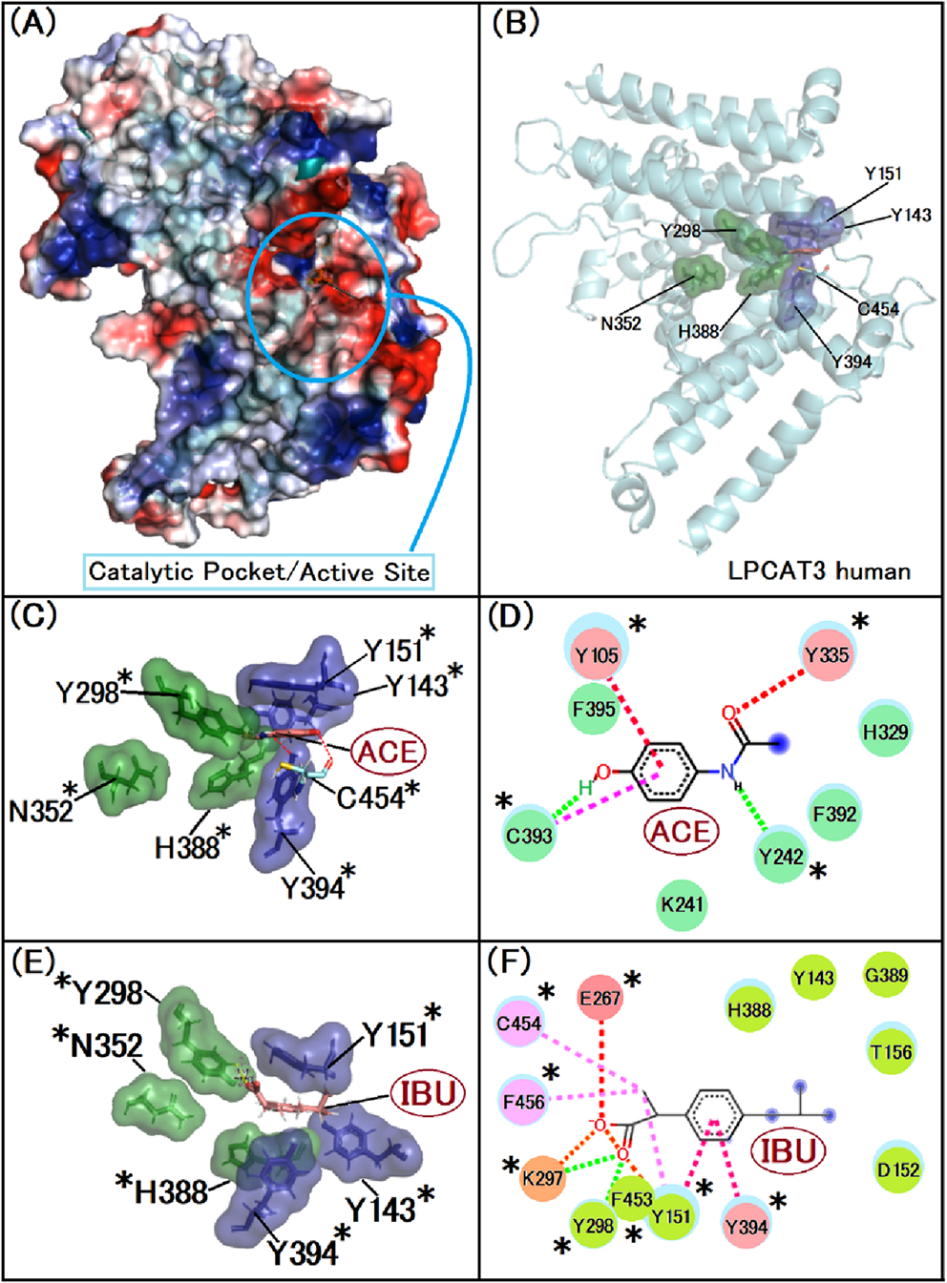
Interactions between NSAIDS and LPCAT3. (A) We display the three-dimensional structure of the native LPCAT3 protein, highlighting the active site cavity. This image was created with Pymol, the surface is colored according to the electrostatic potential. As the color legend indicates, the red color (negative potential) arises from an excess of negative charges near the surface, and the blue color (positive potential) occurs when the surface is positively charged. The white regions correspond to neutral potential. (B) Illustrates the crucial residues involved in the enzymatic activity of LPCAT3. Sections, this image was created with Pymol who show the protein structure in cartoon. The surface of the amino acids in the secondary pocket was represented in purple and the amino acids present in the catalytic triad were in green. (C) The image was created with Pymol, the surface of the amino acids in the secondary site was represented in purple and the amino acids present in the catalytic triad were in green, where in the image the compound is represented in Blue. (D) Depicts the interaction of Acetaminophen (ACE) with the enzyme’s active site. That image was created with Biovia Discovery Studio, following color patterns related to interactions, green for van der Waals, dashed signaling possible hydrogen bond interactions and pink for staked pi interactions, while (E), this image was created with Biovia Discovery Studio, following color patterns related to interactions, green for van der Waals, dashed signaling possible hydrogen bond interactions, orange the pi alkyl and pink for staked pi interactions and (F) show the interaction of Ibuprofen (IBU) with the active site of LPCAT3. Asterisks indicate the important residues of the active site; this image was created with Pymol. The surface of the amino acids in the secondary site was represented in purple and the amino acids present in the catalytic triad were in green, where in the image the compound is represented in rose.

**Table 2:**
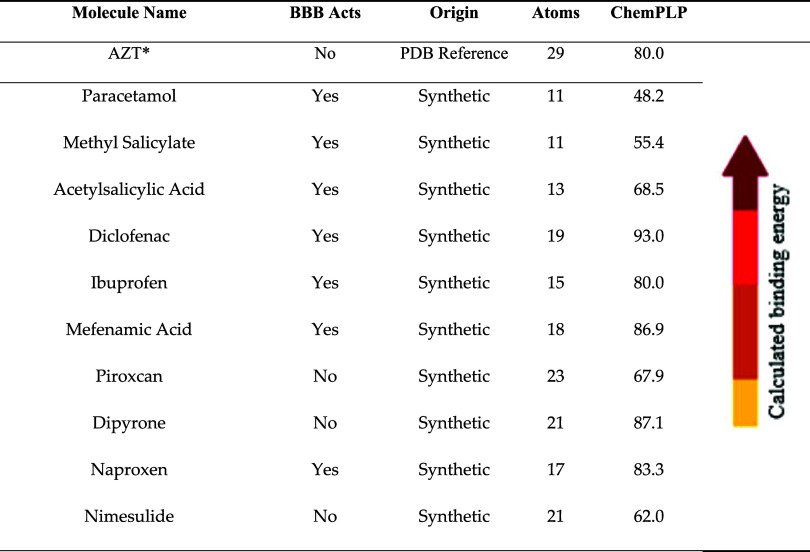
Gold PLP Interaction Analysis of Anti-Inflammatory Drugs with LPCAT3 Protein, Highlighting the Optimal Interaction Scores Achieved with Acetaminophen and Ibuprofen were Scored in Table

* Lysophosphatidyl-choline is a reference inhibitor for the LPCAT3 protein, so a docking was performed to guide the results, a normalized score, dividing the GOLD PLP score by the number of heavy atoms was created for better interaction analysis.^9^

The metabolic implications of LPCAT3 significantly impacts the biology of tumor cells by modifying membrane lipid compositions, thereby affecting crucial cellular processes such as proliferation, migration, and invasion. Modulating LPCAT3 activity or expression can effectively influence membrane dynamics and signaling pathways involved in tumor progression, making LPCAT3 an extremely promising target for cancer therapy.^42^

Pathogenically, LPCAT3 is closely linked to tumors resistance to ferroptosis, a type of lipid peroxide-mediated cell death. Overexpressing LPCAT3 in tumor cells effectively prevents ferroptosis, thereby promoting survival and growth. Conversely, inhibiting LPCAT3 might significantly inhibit tumor growth by sensitizing cells to this form of cell death.^43^

The interaction of anti-inflammatory agents is stabilized by the tyrosine tunnel so that it binds to the Histidine essential for the enzyme activity of the protein.

This already shows us the possibility of physical inhibition of the protein acting with the PUFA’s, meaning that they are unable to stabilize in the site to undergo the enzymatic action of membrane fluid repair carried out by LPCAT3.^42^

The study of human sPLA_2_ was carried out in stages, starting with the cocrystallographic structure available in the PDB (ID: 5OWC), which depicts the enzyme complexed with its natural ligand. Based on this template, molecular docking as we see on Table 3 and molecular dynamics simulations were performed. During this process, structural gaps and errors were identified and corrected by predictive modeling with AlphaFold3, using Uniprot data as a refs 44,45 .

**Table 3:** Gold PLP Interaction Analysis of Anti-Inflammatory Drugs with sPLA2 Protein, Highlighting the Optimal Interaction Scores Achieved with Acetaminophen and Ibuprofen were Scored in Table

molecule name	BBB acts	origin	atoms	ChemPLP
AZT^a^	no	PDB reference	29	80.0
paracetamol	yes	synthetic	11	48.2
methyl salicylate	yes	synthetic	11	55.4
acetylsalicylic acid	yes	synthetic	13	68.5
diclofenac	yes	synthetic	19	93.0
ibuprofen	yes	synthetic	15	80.0
mefenamic acid	yes	synthetic	18	86.9
piroxcan	no	synthetic	23	67.9
dipyrone	no	synthetic	21	87.1
naproxen	yes	synthetic	17	83.3
nimesulide	no	synthetic	21	62.0

a AYZ is a reference inhibitor for the sPLA2 protein, so a docking was performed to guide the results, a normalized score, dividing the GOLD PLP score by the number of heavy atoms was created for better interaction analysis.

Notably, secretory PLA2s are small, calcium-dependent enzymes characterized by a conserved catalytic domain and a distinct hydrophobic surface that facilitates their binding to phospholipid membranes, where substrate hydrolysis occurs. As highlighted by Khosa et al.,^46^ membrane anchoring is a crucial step for enzymatic activation, influencing substrate accessibility and catalytic efficiency.^47,48^

It was observed that the presence of water molecules around the Ca^2+^ ions is essential for the stability of the active structure of the enzyme.^49^ These molecules form a solvent shield that maintains the specific chemical properties of the calcium coordination region, which is essential for the catalytic activity of sPLA2.^50^

The catalytic mechanism and structural features of human sPLA2 have been well documented in the literature, with several residues playing a key role in enzymatic function and substrate recognition. At the heart of the active site are the conserved residue Asp47, His46 and Glu54 which orchestrate the hydrolysis of phospholipid substrates.^51^

Form the core of the catalytic site: Asp47 is directly involved in catalysis, stabilizing Ca^2+^ and facilitating the nucleophilic attack on the ester bond; His46 acts as a general base, essential for the activation of the water molecule involved in the hydrolysis reaction; Glu54 contributes to the geometry of the active site, keeping the calcium ion in position. The cofactor Ca^2+^ stabilizes the tetrahedral intermediate and helps to position the substrate correctly, supported by the set of water molecules structured around it.^47^

The region known as the calcium loop is formed by residues Cys27 and Cys43, which form a desulfated bridge that is crucial for maintaining the three-dimensional structure of the enzyme. Other residues that form the catalytic environment include Gly28, Leu29, Gly30—flexible and located at the entrance of the active site, they participate in the formation of the binding cavity and the coordination of Ca^2+^; Ile2, Leu5, Val9, Met21—form the hydrophobic pocket for the accommodation of the acyl tail of the phospholipid; Pro17—generates a structural twist that allows the formation of the substrate entry site.^46,52–54^

The molecular dynamics analyses revealed striking differences in the interaction profile of NSAIDs with AChE and LPCAT3, indicating potential modulation mechanisms beyond classic cyclooxygenase inhibition. Nimesulide was found to demonstrate a higher degree of affinity for AChE (Δ*G* = −24.20 ± 3.70 kcal/mol), with the presence of significant interactions with essential aromatic and polar residues, including Trp286, Tyr341, Val294, Phe295, Arg296, and Phe297. This pattern suggests simultaneous binding to the central aromatic patch and the peripheral site (P-site), reminiscent of the profile of classic inhibitors, such as donepezil, recognized for its combined competitive and allosteric action as cam be observed at the heatmap on Figure 2.^40,55^

**Figure 2:**
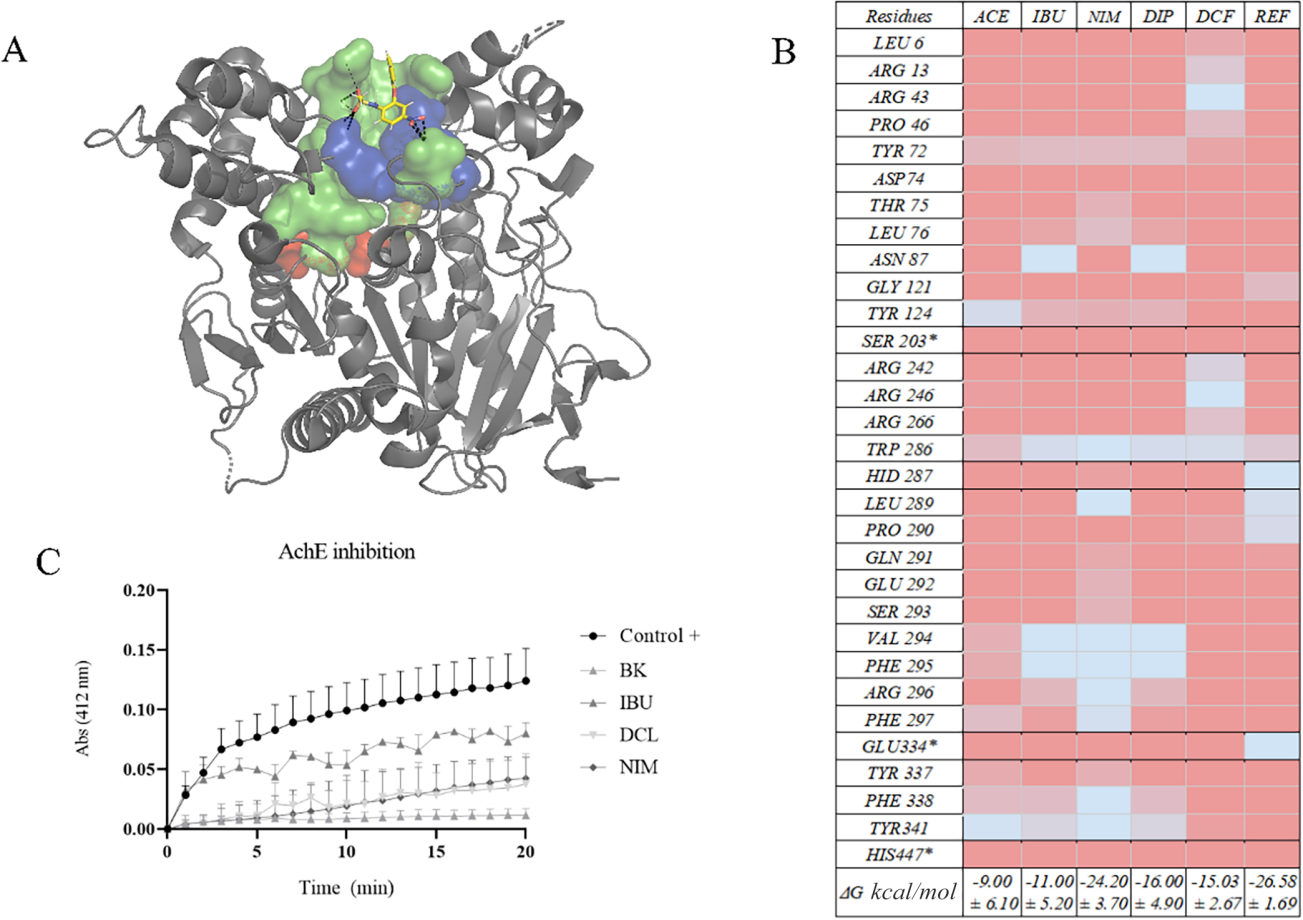
Structural and functional characterization of NSAID interactions with human AchE. (A) Three-dimensional representation of the AchE active site in complex with nimesulide (NIM), residues are categorized based on their functional location within the enzyme: peripheral anionic site (PAS), catalytic site (CAS), and structural or secondary residues (CHANNEL). Image generated with PyMOL. (B) Heatmap of per-residue binding free energy decomposition (Δ*G*, kcal/mol) estimated by MM-GBSA, representing contributions to complex stability. Blue indicates favorable interactions (negative Δ*G*), while red denotes unfavorable contributions (positive Δ*G*). (C) Enzymatic assay of AchE activity.

Furthermore, the enzymatic assays utilized provide solely an initial assessment of inhibitory activity, neglecting to consider factors such as metabolism, bioavailability, permeability through the blood-brain barrier, and potential adverse systemic effects.

As reported in the extant literature, the findings of earlier studies indicate that effective AChE inhibition is characterized by stable binding at the catalytic site and interactions at the P-site, which modulate enzymatic activity by prolonging acetylcholine availability and activating the anti-inflammatory cholinergic axis.^40,56^ The present study corroborates this trend, highlighting the potential of nimesulide to act as a dual modulator, thereby expanding the understanding of NSAID functionality beyond the scope of simple COX inhibition. In contrast, compounds such as diclofenac and ibuprofen exhibited intermediate affinities and mechanisms based predominantly on electrostatic or hydrophobic interactions, as described in studies such as,^26,57^ reflecting lower cholinergic specificity.

The energy breakdown by residue further reinforces the importance of aromatic and charged residues in the stability of nimesulide binding, pointing to mechanisms that may combine competitive inhibition at the active site with allosteric modulation via the peripheral site. This finding aligns with mechanistic models elucidated in the extant literature, wherein interaction with the P-site modifies the dynamics of the AChE access tunnel, thereby regulating its activity and exerting an influence on neuroinflammatory processes. However, it is imperative to acknowledge the limitations intrinsic to in silico studies. These limitations include the inability to fully replicate the complexity observed in vivo and the reliance on crystallographic conformations utilized for simulations, which can introduce structural biases.

It is acknowledged that the primary affinity of NSAIDs remains for COX, and that this characteristic poses a challenge for repurposing. However, the objective of this work was not to propose a direct replacement for classic AChE inhibitors, but rather to explore the potential multitarget effects of these compounds. The precise therapeutic dosage required to achieve significant AChE inhibition remains to be investigated in cellular and in vivo models. This limitation was already highlighted in the revised text, where we emphasized that the results presented constitute initial mechanistic hypotheses that require further validation. With regard to the selectivity of CNS drugs, it should be noted that certain NSAIDs exhibit partial permeability to the blood-brain barrier. However, it is also important to acknowledge the significant role of AChE in peripheral tissues, a factor which serves to expand the applicability of these findings beyond the confines of the CNS.

With regard to LPCAT3, the most significant affinity for nimesulide (Δ*G* = −37.60 ± 4.90 kcal/mol) indicates a potential pleiotropic effect, on Figure 3, involving stabilization at crucial catalytic sites (residues Cys301/Trp302, Thr355, Asn352, Trp387) as we see on the Figure 4, consistent with its role in lipid remodelling and regulation of metabolic inflammation. This finding unveils an additional mechanism through which nimesulide may modulate metabolic pathways associated with insulin resistance and obesity, thereby extending therapeutic repositioning to interconnected metabolic and neuroinflammatory conditions.

**Figure 3:**
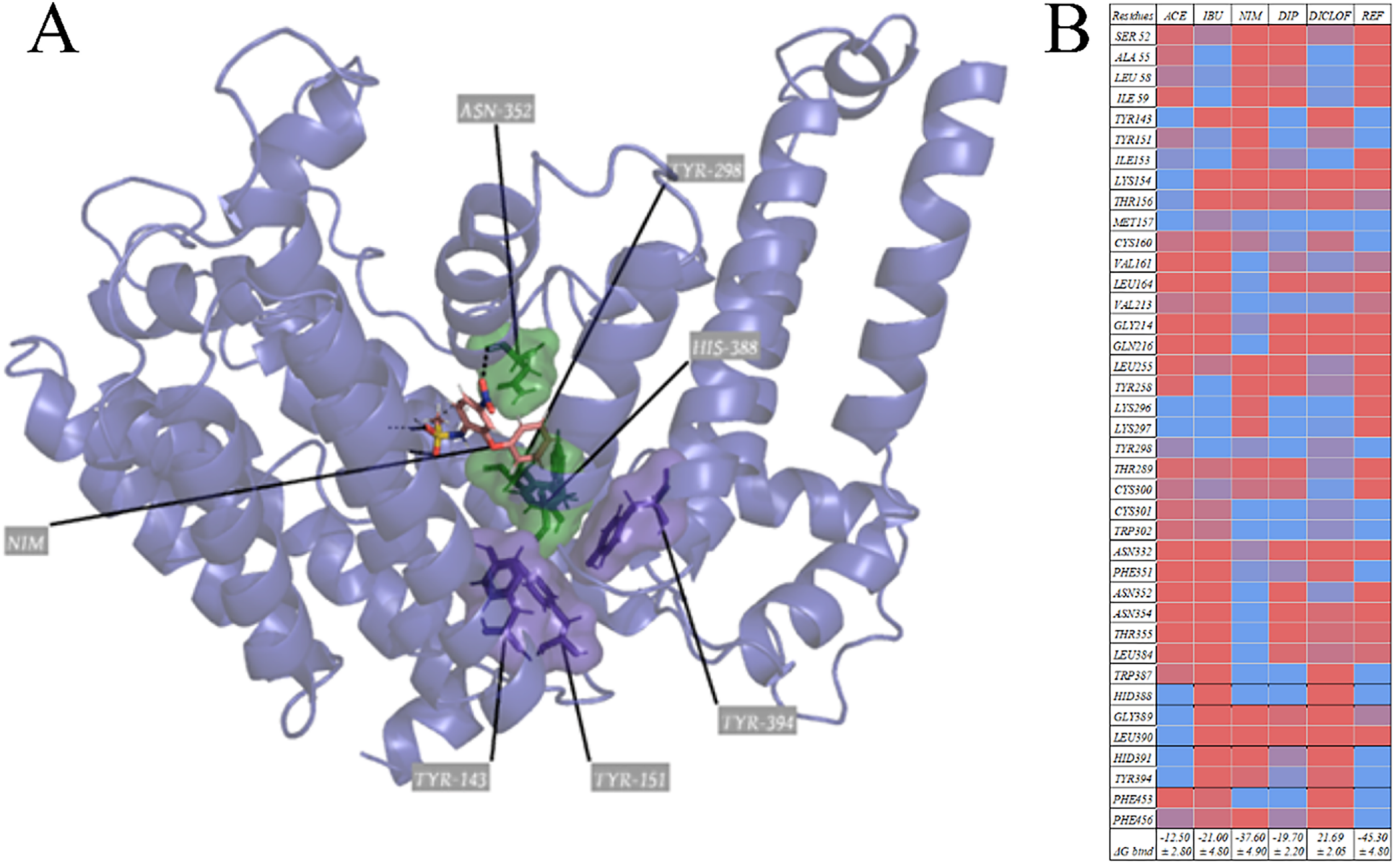
Illustrates in detail the interaction sites and dominant forces between nimesulide (NIM) and LPCAT3, captured at frame 150 ns of the molecular dynamics simulation. (A) The catalytic site is highlighted, with the protein surface represented in purple and the reactive residues shown in green. (B) In parallel, a heatmap depicts the binding free energy (Δ*G*) estimated by MM-GBSA for ligands complexed to the LPCAT3, including per-residue resolution (mean ± SD). The energy contributions of the most relevant residues to complex stability are represented by color intensity, with blue indicating more favorable (negative Δ*G*) interactions and red indicating less favorable (positive Δ*G*) interactions.

**Figure 4:**
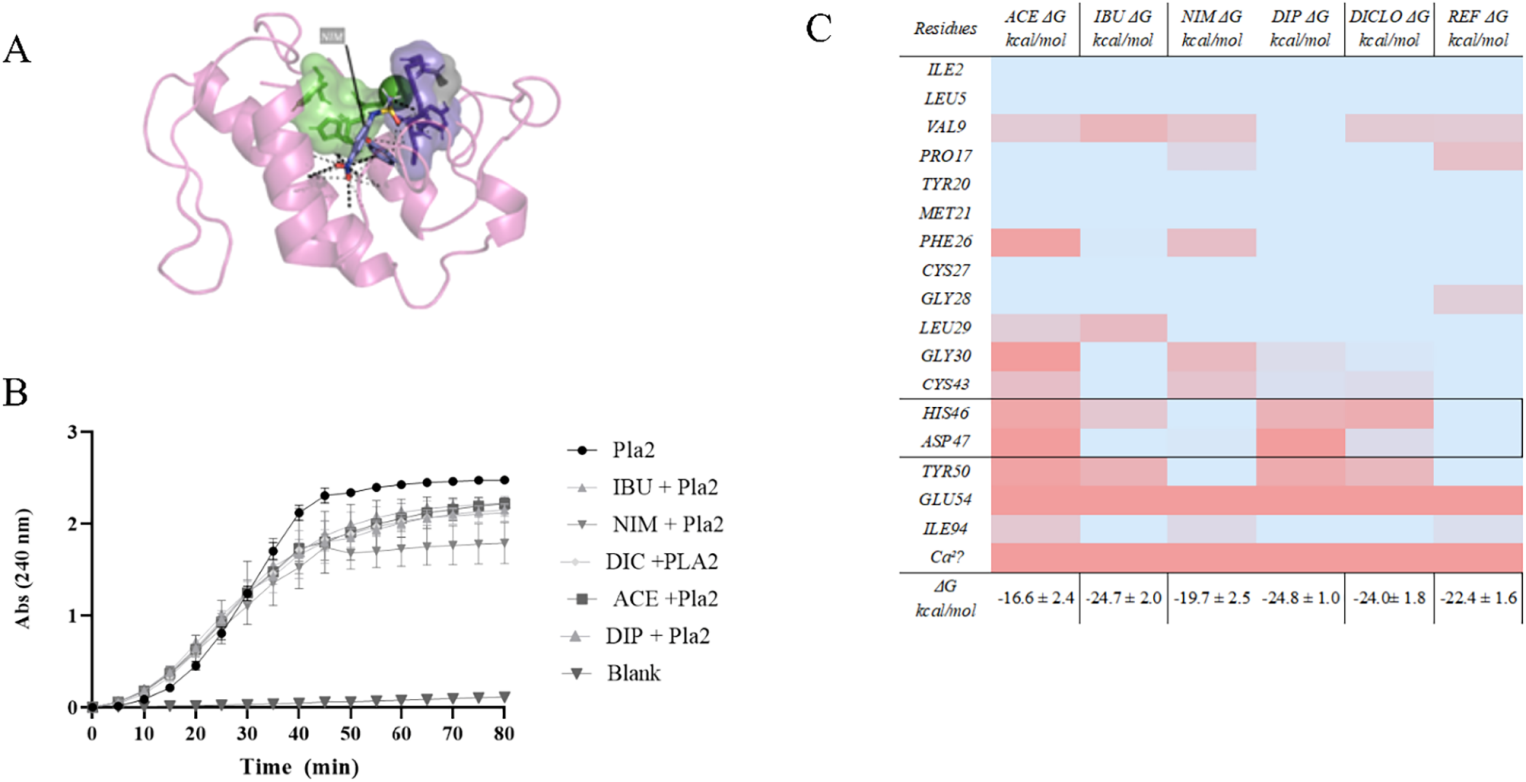
Structural and functional characterization of NSAID interactions with human sPLA_2_ (group IIA). (A) Three-dimensional representation of the sPLA_2_ active site in complex with nimesulide (NIM), showing the catalytic Ca^2+^ loop (green), hydrophobic pocket (purple surface), and ligand position. Image generated with PyMOL. (B) Enzymatic assay of sPLA_2_ activity in the presence of different NSAIDs compared with untreated control, highlighting the time-dependent inhibitory effect of ibuprofen (IBU), dipyrone (DIP), and nimesulide (NIM), as well as the weaker inhibition by diclofenac (DCF) and acetaminophen (ACE). (C) Heatmap of per-residue binding free energy decomposition (Δ*G*, kcal/mol) estimated by MM-GBSA, representing contributions to complex stability. Blue indicates favorable interactions (negative Δ*G*), while red denotes unfavorable contributions (positive Δ*G*).

In terms of therapeutic implications, the inhibition of AChE by nimesulide suggests a potential role in neurodegenerative diseases such as Alzheimer’s and Parkinson’s, where there is a compromise to the cholinergic pathway. However, a crucial point is the permeability of the blood-brain barrier (BBB). Pharmacokinetic studies indicate that nimesulide has good penetration into the central nervous system, although effective in vitro concentrations should be carefully compared with therapeutic plasma concentrations to assess clinical viability. Furthermore, potential off-target effects and cholinergic toxicities, such as those seen with selective AChE inhibitors, should be further investigated to mitigate associated risks.^58–60^

Despite the predicted high affinity for AChE and sPLA_2_, nimesulide presents significant clinical limitations due to its hepatotoxicity profile, a fact that has led to regulatory restrictions in several countries. Consequently, while the compound was maintained in the study as a reference interaction, its clinical application should be considered with caution. Future repositioning strategies may benefit from structural analogs or nimesulide derivatives with more favorable safety profiles.

Notwithstanding the encouraging outcomes, it is imperative to underscore the methodological limitations intrinsic to the present study. The *in silic*o approach, despite its robustness and acceptance for predicting molecular interactions, is inadequate in fully capturing the biological and physiological complexity found in living systems.

The human sPLA_2_ molecular dynamics simulations revealed differential binding modes when compared with the reference inhibitor AZT. Per-residue energy decomposition (MM-GBSA) highlighted key contributions from residues of the catalytic loop (His46, Asp47, Glu54) and the calcium-binding region (Cys27, Gly28, Leu29, Gly30), on Figure 4.

Nimesulide (Δ*G* = −19.7 ± 2.5 kcal/mol) demonstrated favorable stabilization, with strong contributions from ILE2, LEU29, and HIS46, partially mimicking the canonical inhibition mode of AZT (REF) (Δ*G* = −22.4 ± 1.6 kcal/mol). Diclofenac (Δ*G* = −24.0 ± 1.8 kcal/mol) interacted with LEU29 and ASP47 but showed greater fluctuations in binding energy, suggesting lower stability.

Unexpectedly, case of IBU, the calculated binding energy was (Δ*G* = −24.7 ± 2.0 kcal/mol), a value that would initially suggest good affinity^61^ and dipyrone (Δ*G* = −24.8 ± 1.0 kcal/mol) outperformed REF in terms of binding energy. Ibuprofen stabilized through interactions with CYS27, GLY30, and ASP47, while dipyrone anchored at GLY28 and LEU29. These patterns suggest alternative competitive binding within the catalytic groove, although with potential destabilization of Ca^2+^ coordination. In contrast, acetaminophen (Δ*G* = −16.6 ± 2.4 kcal/mol) exhibited only modest affinity, correlating with its limited inhibitory profile.

The positive contributions of Ca^2+^ binding energy in all complexes reinforced the hypothesis of partial electrostatic repulsion or misalignment of the coordination network. Ibuprofen and dipyrone significantly altered Ca^2+^ stabilization (29.46 and 20.11 kcal/mol, respectively) compared to REF (32.61 kcal/mol), consistent with allosteric interference with cofactor alignment and substrate positioning. This finding suggests that a favorable binding energy does not necessarily guarantee functional inhibitory activity, particularly in cases where the essential cofactor is destabilized as Gaillard and Simonson.^62^

This outcome stands in contrast to the prevailing hypothesis that ibuprofen would interact preferentially with the catalytic site. As a NSAID with a carboxylic group and hydrophobic characteristics, a direct binding with catalytic residues was hypothesized. However, the data suggest that ibuprofen interacts with alternative regions–possibly hydrophobic interfaces–accessible only in the oligomeric form, a pattern similar to that reported by Salvador et al., who observed inhibition by occupation of peripheral sites.^63,64^

We can improve that which the enzymatic activity assays corroborated the computational predictions. Ibuprofen and dipyrone markedly reduced sPLA_2_ hydrolytic activity in a time-dependent manner, displaying inhibitory profiles close to AZT. Nimesulide showed moderate inhibition, consistent with its intermediate ΔG. Diclofenac and acetaminophen produced weaker inhibition, with activity curves converging toward the untreated control after prolonged incubation. Importantly, ibuprofen and dipyrone showed potent inhibitory activity, despite not being traditionally associated with sPLA_2_ inhibition, indicating an unanticipated off-target mechanism.

Taken together, the results demonstrate that common NSAIDs—particularly ibuprofen and dipyrone—possess previously underappreciated inhibitory potential against human sPLA_2_, in some cases comparable to AZT. While nimesulide showed intermediate but stable inhibition, diclofenac and acetaminophen presented weaker binding and activity. The destabilization of Ca^2+^ coordination by ibuprofen and dipyrone suggests an atypical mode of inhibition, possibly involving allosteric or peripheral binding sites rather than exclusive targeting of the catalytic triad.

These findings extend the pharmacological profile of NSAIDs beyond COX inhibition, highlighting their pleiotropic potential in regulating enzymes central to lipid mediator homeostasis. Such modulation may contribute both to their therapeutic effects in inflammation and to off-target activities in cardiovascular and neurodegenerative contexts. Furthermore, the results support the rationale for repurposing selected NSAIDs as dual modulators of phospholipase activity, with potential applications in conditions associated with lipid metabolism imbalance and uncontrolled inflammation.

Consequently, the conclusions presented here should be interpreted as well-founded hypotheses that require validation and extension through cellular studies and in vivo animal models to confirm their therapeutic relevance and clinical transposition. In summary, the present study lends further support to the hypothesis that certain NSAIDs, notably nimesulide, manifest multiple modes of interaction with AChE and LPCAT3, thereby suggesting the presence of allosteric/peripheral mechanisms in addition to classical inhibition. The integration of computational and biochemical approaches provided a valid framework for future in vitro and in vivo research. This will potentially enable new pharmacological repositioning of these molecules in neurodegenerative, metabolic, and inflammatory diseases. Further functional validation in cellular and animal models is imperative to confirm the physiological and therapeutic relevance of these findings and to overcome limitations inherent in computational models.

## 4 Conclusions

This study demonstrated that selected NSAIDs, particularly nimesulide, exhibit marked affinity and inhibitory potential toward AChE, thus indicating a pharmacological role for these compounds that extends beyond classical cyclooxygenase inhibition. Through in silico analyses, including molecular docking, molecular dynamics simulations, and MM-GBSA free energy estimations, NSAIDs were shown to establish stable interactions with AChE, LPCAT3, and PLA2. These three molecular targets are integral to the processes of neuroinflammation, and lipid remodelling associated with obesity. Among the compounds that were tested, nimesulide demonstrated the highest binding affinity, distinguishing itself as a potential dual-acting modulator with translational relevance.

From a pathophysiological perspective, the inhibition of AChE by nimesulide leads to an increase in acetylcholine bioavailability, resulting in the subsequent activation of the cholinergic anti-inflammatory pathway. This mechanism contributes to the modulation of microglial activation, suppression of pro-inflammatory cytokine release, and protection against oxidative stress. All of these are central to the maintenance of neuronal integrity in conditions such as Alzheimer’s disease and Parkinson’s disease.

Nevertheless, the potential modulation of AChE by NSAIDs is likely to occur in a dose- and tissue-dependent manner, and its physiological significance remains to be fully validated. With regard to its toxicological profile, nimesulide has well-documented limitations, including hepatotoxicity, which have led to clinical restrictions. Its inclusion in the present analysis was intended primarily as a molecular reference for NSAID–AChE interactions rather than as a direct repurposing candidate. Interestingly, nimesulide has also been reported to reduce BBB disruption and leukocyte infiltration in models of cerebral ischemia, thereby highlighting an indirect effect on the brain environment.^36^ These findings are consistent with the hypothesis that certain NSAIDs can influence central pathways either by partial BBB penetration or by indirect peripheral mechanisms.

In summary, the results demonstrate that NSAIDs can modulate not only cyclooxygenases but also alternative molecular targets such as AChE, LPCAT3, and sPLA_2_, expanding their pharmacological relevance in the context of inflammation and neurodegeneration. The observed modulation of LPCAT3 and PLA_2_ suggests an additional contribution of NSAIDs to lipid remodelling within the Lands cycle, whose disruption is associated with membrane rigidity, insulin resistance and adipose tissue inflammation. The demonstrated interactions of nimesulide with key residues in these enzymes reinforce its potential role in mitigating metabolic dysfunctions characteristic of obesity and metabolic syndrome.

Taken together, these findings provide a foundation for the investigation of NSAIDs as multitarget modulators in neuroinflammatory and metabolic contexts. However, they should be regarded as mechanistic hypotheses requiring rigorous validation in dose–response, cellular and in vivo studies. Particular caution is warranted in the case of nimesulide due to its adverse safety profile, which highlights the need to explore safer structural analogues with similar binding properties.
